# HLA-DR-Positive NK Cells Expand in Response to Mycobacterium Tuberculosis Antigens and Mediate Mycobacteria-Induced T Cell Activation

**DOI:** 10.3389/fimmu.2021.662128

**Published:** 2021-05-03

**Authors:** Sofya A. Kust, Maria A. Streltsova, Alexander V. Panteleev, Natalya L. Karpina, Irina V. Lyadova, Alexander M. Sapozhnikov, Elena I. Kovalenko

**Affiliations:** ^1^ Laboratory of Cell Interactions, Shemyakin-Ovchinnikov Institute of Bioorganic Chemistry of Russian Academy of Science, Moscow, Russia; ^2^ Laboratory of Biotechnology, Central Tuberculosis Research Institute, Moscow, Russia; ^3^ Diagnostic Outpatient Department, Central Tuberculosis Research Institute, Moscow, Russia; ^4^ Laboratory of Cellular and Molecular Basis of Histogenesis, Koltzov Institute of Developmental Biology of the Russian Academy of Sciences, Moscow, Russia

**Keywords:** NK cells, antigen presentation, Mycobacterium tuberculosis, IFNγ, CD4^+^ T cells

## Abstract

NK cells play an important role in the control of tuberculosis infection: they are not only able to kill the infected cells, but also control the activity of macrophages and development of the adaptive immune response. Still, there is little information on the role of specific NK cell subsets in this network. In this study, we focused on the mycobacteria-driven responses of the NK cells expressing HLA-DR – a type of MHC class II. We have revealed that this subset is increased in the peripheral blood of patients with primary diagnosed tuberculosis, and expands in response to *in vitro* stimulation with ultrasonically destroyed *Mycobacterium tuberculosis* cells (sonicate). The expanded HLA-DR^+^ NK cells had less differentiated phenotype, higher proliferative activity and increased expression of NKp30 and NKp46 receptors. HLA-DR^+^CD56^dim^ NK cells showed higher IFNγ production and degranulation level than the respective HLA-DR^−^ NK cells in response to both 24 h and 7 day stimulation with sonicate, while HLA-DR^+^CD56^bright^ NK cells mostly demonstarted similar high responsiveness to the same stimulating conditions as their HLA-DR^−^CD56^bright^ counterparts. After preliminary incubation with destroyed mycobacteria, cytokine-activated HLA-DR-expressing NK cells were able to mediate mycobacteria-induced and HLA-DR-dependent cytokine production in autologous CD4^+^ T cells. Thus, functionally active HLA-DR^+^ cells seem to be one of the NK cell subsets providing an important link to the adaptive immunity.

## Introduction

More than a third of the Earth’s population is infected with *Mycobacterium tuberculosis (Mtb)*; 8.9–11.0 million people develop tuberculosis annually, and 1.3-1.5 million of cases are fatal (WHO, 2020). However, due to the coordinated actions of the innate and adaptive immune systems, most of the infected people do not develop clinical signs of tuberculosis disease. A dynamic equilibrium is established in these individuals when bacteria persist in the lungs in a dormant state in granulomas as long as the immune system is able to control them (1). Cellular immune response plays a great role in the protection against tuberculosis (TB), mainly *via* the IL-12-Th1-IFNγ-dependent pathway involving macrophages (2). Recently, however, it has become clear that many other immune cell subsets contribute to the effective immune response against *M. tuberculosis*, including NK cells (3). NK cells are not only able to kill the infected cells, but they are also important as a link between the innate and adaptive immunity, producing cytokines and affecting the functions of other immune cells (3).

It was shown that NK cells are able to recognize mycobacterial cells directly. Stimulation with Bacillus Calmette-Guerin (BCG) induced the expression of activation markers, increase in cytotoxic activity and IFNγ production in NK cells from healthy donors in the absence of monocytes/macrophages in the system (4–6). Further studies showed that, most likely, NK cells interact with the components of the mycobacterial cell wall through the activating receptors NKp44 and TLR2 (7, 8). However, the level of IFNγ response of NK cells to the presence of mycobacteria varied greatly between donors (6, 8), which may correlate with the KIR receptor haplotype (9).

NK cells can positively and negatively influence the activity of various T cells subsets responding to tuberculosis infection. After removal of NK cells from PBMCs of healthy donors, the proliferation of γδ T cells in response to *M. tuberculosis* antigens was greatly reduced (10). Similar results were obtained after removing NK cells from PBMCs of healthy donors responding to tuberculin: CD8^+^ T cells lysed *M. tuberculosis*-infected monocytes less effectively, and the total number of *M. tuberculosis*-specific IFNγ-producing CD8^+^ T cells was also decreased (11). It turned out that IFNγ produced by NK cells was needed to trigger the secretion of IL-15 and IL-18 by monocytes, which, in turn stimulated the proliferation of specific CD8^+^ T cells.

At the moment, very few works are devoted to the interaction of NK cells with CD4^+^ T cells during tuberculosis infection, while there is evidence that these subsets can communicate in the course of the immune response through reciprocal stimulation with cytokines and intercellular contacts (12). For instance, the production of IFNγ by NK cells can be essential for driving Th1 differentiation of naïve CD4^+^ T cells (13, 14). Of especial interest is an activated NK cell subset expressing HLA-DR – a type of MHC class II, as they can potentially interact directly with TCR-CD4 complex and usually display increased IFNγ production (15, 16). Moreover, high numbers of HLA-DR^+^ NK cells are found in the infected lungs of TB patients (17, 18). HLA-DR-expressing NK cells obtained *in vitro* under cytokine stimulation were shown to stimulate nonspecific activation and partial differentiation of CD4^+^ T cells (19). Several studies demonstrated the ability of HLA-DR^+^ NK cells to stimulate specific T cell response to certain antigens, similarly to professional antigen-presenting cells (APCs): tetanus toxin and Der pI house dust mite allergen (20), HSV particles and peptides isolated from them (21), HCMV particles and HCMV-antibody complex  (22). As HLA-DR-positive NK cells were shown to accumulate in *M. tuberculosis* infected lungs *in vivo* (17, 18) and expand in response to BCG stimulation *in vitro* (6), they might play a role in the processing and presentation of mycobacterial antigens as well.

In this work, we have demonstrated the expansion of HLA-DR^+^ NK cells, predominantly CD56^bright^, in the blood of primary tuberculosis patients comparing to healthy donors. We characterized the HLA-DR-expressing NK cell response to destroyed *M. tuberculosis in vitro* both in isolated cultures and in the context of peripheral blood mononuclear cells (PBMC): their phenotypic and functional properties, proliferative activity, resistance to mycobacteria-mediated suppression of functions. Besides, we have shown the ability of HLA-DR^+^ NK cells to induce Mtb-specific and HLA-DR-dependent CD4^+^ T cell activation in a Th1-like manner.

## Materials and Methods

### Subjects and Ethics Statement

Blood samples were obtained from 46 volunteer healthy adults and 22 tuberculosis patients. TB patients were recruited from the Central Tuberculosis Research Institute (Moscow, Russia), had primarily diagnosed TB and have not received anti-tuberculosis therapy before the study. All the participants gave written informed consent. The study was approved by the local ethics committee of the Russian State Medical University (protocol #169 from 20.11.2017) and of the Central Tuberculosis Research Institute of Russian Academy of Sciences (IRB #1), and was conducted in accordance to the principles expressed in the Helsinki Declaration.

### Isolation of Cell Subpopulations

PBMC fraction was obtained by gradient centrifugation of blood samples using a standard Ficoll solution (PanEco, Russia), density 1.077. NK cells and CD4^+^ T cells were isolated from PBMC using NK cell Isolation Kit (Miltenyi Biotec, Germany) and MojoSort™ Human CD4 T Cell Isolation Kit (Biolegend, USA) by negative magnetic separation, according to the manufacturers’ protocols. NK cell and T cell purity reached 95-99%. Lymphocytes were cultivated in NK MACS medium (Miltenyi Biotec, Germany) supplemented with 10% FCS (HyClone, USA) and Antibiotic Antimycotic Solution (Sigma-Aldrich, USA).

Dendritic cells were obtained based on the standard protocol (23). Briefly, PBMC were plated at a concentration of 10^6^ cells/ml in RPMI-1640 medium (PanEco, Russia) supplemented with 10% FCS (HyClone, USA), 2 mmol/L of L-glutamine (PanEco, Russia) and Antibiotic Antimycotic Solution (Sigma-Aldrich, USA) for 24h, then gently washed from non-adherent cells and cultured for 10 days. IL-4 (50 ng/ml) and GM-CSF (60 ng/ml) (Sci-Store, Russia) were added at day 0, day 1 and then every 3 days, with the fresh medium. At day 10, more than 90% of cells had CD14^low^CD11c^+^HLA-DR^hi^CD86^hi^ phenotype.

### Flow Cytometry

Cells were labeled with the following mouse anti-human monoclonal antibodies: СD56-APC, CD56-PE, CD3-FITC, HLA-DR-PE-Cy7 (Beckman Coulter, USA), CD8-PE-Cy5 (BD Bioscience, USA), CD57-PE, NKG2D-PE (eBioscience, USA), CD3-PE (Dako, Denmark), NKG2C-AF488, NKG2C-PE (R&D Systems, USA), KIR2DL2/DL3-PE, CD16-VioGreen, CD14-PE-Cy7б CD4-APC (Miltenyi Biotec, Germany), NKp30-FITC, CD86-PE (Biolegend, USA), HLA-DR-FITC, HLA-DR-PE, HLA-DR-Brilliant Violet 421, CD56-PE-Cy7, CD3-APC, NKp44-PE, NKp46-PE-Cy7, CD56-Brilliant Violet 421 (Sony Biotechnology, USA). Detailed information on the clones and isotypes of the antibodies can be found in the **Supplementary Table 1**. CD7 staining was performed with hybridoma supernatant, kindly provided by Prof. Miguel Lopez-Botet (University Pompeu Fabra, Barcelona, Spain). SytoxRed live/dead stain was used to exclude dead NK cells. Before measurement, cells from patients with tuberculosis were fixed with 1% paraformaldehyde solution (Carl Roth, Germany) for 24 h.

For intracellular staining of IFNγ and TNFα, 10 μg/ml of brefeldin A (AppliChem, Germany) was added to the cell culture 4h before measurement. After that, cells were labeled for surface markers, washed, and then fixed and permeabilized with Cytofix/Cytoperm solution (BD Biosciences, USA). The required amount of antibodies against IFNγ and/or TNFα (IFNγ-FITC, IFNγ-PE, TNFα-FITC, Miltenyi Biotec, Germany) was added in Perm/Wash solution (BD Biosciences, USA) for 30 min on ice, then cells were washed and analyzed.

Cells were analyzed on a FACSCalibur flow cytometer (Beckton Dickinson, USA) using the CellQuest software, or on a MACSQuant10 flow cytometer (Miltenyi Biotec, Germany) using the manufacturer’s software. The data obtained were processed and presented using FlowJo X 10.0.7r2 (FlowJo LLC, Ashland, OR, USA).

### Analysis of Proliferative Activity

NK cell proliferation was assessed using the fluorescent dye CFSE. Freshly isolated NK cells were stained with CFSE at a concentration of 5 μmol/L (eBioscience, USA), then washed and transferred to the medium with the necessary stimuli. The percentage of cells that have reduced or lost CFSE fluorescence was measured by flow cytometry at day 7.

### Degranulation Assay

NK cells were incubated with K562 target cells at 2:1 ratio for 2 h in the presence of 10 μg/ml of monensin and anti-CD107a mAb (VioBlue, Miltenyi Biotec, Germany). After that cells were harvested, stained for surface antigens and analyzed by flow cytometry.

### PBMC and NK Cell Stimulation With *M. tuberculosis* Sonicate

Suspension of ultrasonically destroyed *M. tuberculosis* (sonicate) was subjected to ultrasonic treatment for 20 min to destroy possible aggregates before being used in the experiments. PBMC or isolated NK cells were transferred to full RPMI or NK MACS medium, respectively, at a concentration of 1.5*10^6^ cells/ml, then cultured in different conditions.

For 24 h stimulation, sonicate was added in a concentration of 5 μg/ml for PBMC, and 0.05-10 μg/ml for isolated NK cells. The medium for isolated NK cells was supplemented with 20 ng/ml IL-12 (BD Biosciences, USA) and 20 ng/ml IL-15 (Sigma-Aldrich, USA) for IFNγ production tests and with 500 U/ml IL-2 (Hoffmann-La-Roche, Switzerland) for degranulation tests.

For 7-day stimulation, IL-2 (100 U/ml) or IL-2 (100 U/ml) + sonicate (2 μg/ml) were added to isolated NK cells. The medium supplemented with IL-2 was changed at day 3. At day 7, the cells were transferred to the new medium and left for 24 h (rest period), then re-stimulated with IL-12+IL-15 (20 ng/ml each) for IFNγ production tests and with 500 U/ml IL-2 for degranulation tests.

In a series of experiments, NK cells were first stimulated with IL-2 (100 U/ml) + IL-21 (50 ng/ml) (Biolegend, USA) for 6 days, then left for a rest for 24 h, and re-stimulated with sonicate (2 μg/ml) supplemented with IL-12+IL-15 (20 ng/ml each) to assess IFNγ production or 500 U/ml IL-2 to assess degranulation activity.

### Co-Cultivation of CD4^+^ T Cells With NK Cells and Dendritic Cells

NK cells, pre-activated for 10 days with IL-2 (100 U/ml) + IL-21 (50 ng/ml) + IL-18 (50 ng/ml) (R&D systems, USA), and dendritic cells derived from PBMC were transferred to new growth medium and incubated for 24 h in the presence of *M. tuberculosis* sonicate (2 μg/ml). Then cells were washed and added to CD4^+^ T cells in the ratio T:NK = T:DC = 2:1, in full NK MACS medium supplemented with brefeldin A (10 μg/ml) and Fc-receptor blocker (5 μl for every 10^7^ cells) (Miltenyi Biotec, Germany). Neutralizing antibody against HLA-DR (Sony Biotechnology, USA) was added to some samples. After 20 h of incubation, staining of surface markers and intracellular staining for IFNγ and TNFα was performed, and cells were analyzed by flow cytometry.

### Statistical Analysis

Statistical significance of the differences in the data with normal distribution was determined by paired or unpaired Student’s t-tests and by two-way ANOVA. For non-normally distributed data Mann-Whitney U-test was used. P < 0.05 was considered significant.

## Results

### HLA-DR-Positive NK Cell Subset Is Increased in the Blood of Patients With Tuberculosis

**Figure 1:**
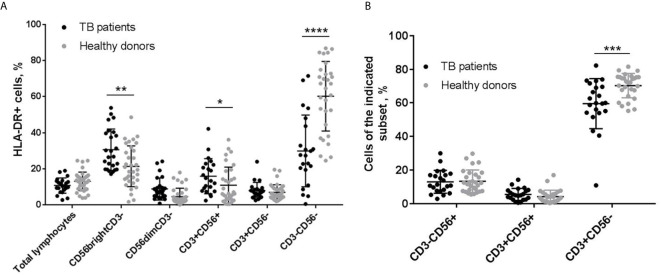
**|** Analysis of PBMC subsets from patients with tuberculosis (TB) and healthy donors. **(A)** Proportions of HLA-DR-expressing cell in total lymphocytes, CD3^−^CD56^bright^, CD3^−^CD56^dim^, CD3^+^CD56^+^, CD3^+^CD56^−^, and CD3^−^CD56^−^ subsets (n = 22 for TB patients and n = 38, 38, 34, 38, 32 for healthy donors, respectively). **(B)** Proportions of NK cells (CD3^−^CD56^+^), and CD56-positive and negative T cells (CD3^+^CD56^+^ and CD3^+^CD56^−^, respectively). N = 22 for TB patients and n=29 for healthy donors. **(A, B)** Individual values are shown together with mean ± SD. Statistical difference was evaluated by standard t-test for data with normal distribution and by Mann-Whitney U-test for data with abnormal distribution. *p < 0,05; **p < 0,01, ***p < 0,001, ****p < 0,0001.

First of all, to estimate the physiological significance of HLA-DR-positive NK cells *in vivo* during tuberculosis infection, we compared the expression of HLA-DR in PBMC subsets isolated from the blood of tuberculosis patients and healthy volunteers. In tuberculosis patients, the proportion of HLA-DR-expressing cells among CD56^bright^ NK cells (CD56^bright^CD3^−^) and NKT-like cells (CD56^+^CD3^+^), was significantly higher compared to healthy donors (**Figure 1A**). According to the literature, infiltration with HLA-DR^+^CD56^bright^ NK cells is also observed in the TB-infected lungs (17, 18). In addition, the level of HLA-DR^+^ NK cells correlated with the level of HLA-DR^+^ NKT-like cells (r=0,566, p=0,006). In a cohort of healthy donors, some people also had a high level of HLA-DR^+^ cells both in CD56^bright^CD3^−^ and CD56^+^CD3^+^ subsets (**Figure 1A**). Expansion of HLA-DR-expressing cells be caused by an active immune response against other pathogens, including latent, as it is shown for adaptive HCMV-associated NK cells (22).

According to our data, tuberculosis patients also had a reduced content of HLA-DR^+^ cells in the CD3^−^CD56^−^ fraction (in healthy donors, this fraction is mainly represented by B cells) (**Figure 1A**) and a reduced proportion of CD3^+^CD56^−^ T cells in the lymphocyte gate (**Figure 1B**), which generally indicates a depressed state of the adaptive immune system. In such conditions, we can suppose that HLA-DR-positive NK cells will play an important role in the development of the immune response against *M. tuberculosis*, possibly, compensating for the deficiencies of the adaptive link. On the basis of these data, we made an assumption for subsequent experiments that HLA-DR-positive NK cells may be responsive to certain mycobacterial antigens. Ultrasonically destroyed *M. tuberculosis* (sonicate) was used as a model mixture of antigens to study NK cell reactions to the presence of mycobacteria.

### Functional Response of HLA-DR^+^ NK Cells to Stimulation With *M. tuberculosis* Antigens

To assess the functional response of HLA-DR-positive and negative NK cells to *M. tuberculosis*, intracellular IFNγ production was measured after stimulation with mycobacterial sonicate. The experiments were carried out using PBMC cultures from healthy volunteers and patients with tuberculosis and cultures of isolated NK cells from healthy volunteers.

**Figure 2:**
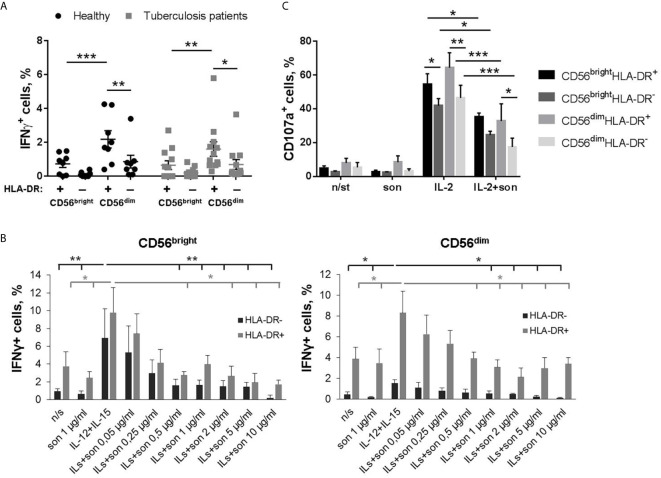
**|** Functional response of NK cells *ex vivo* to 24-hour stimulation with M. tuberculosis sonicate. **(A)** Production of IFNγ by HLA-DR^+^ and HLA-DR^–^ NK cells in PBMC cultures from tuberculosis patients (n = 9) and healthy donors (n = 8) in response to 5 μg/ml sonicate stimulation. Individual values are shown together with Mean ± SEM. Statistical difference was evaluated by two-way ANOVA. **(B)** Production of IFNγ by NK cells after 24 h incubation with IL-12+IL-15 and sonicate in the indicated concentrations, in the CD56^bright^HLA-DR^+^, CD56^bright^HLA-DR^–^, CD56^dim^HLA-DR^+^ and CD56^dim^HLA-DR^−^ subsets. Data are mean ± SEM of four independent experiments. Statistical difference was evaluated by paired t-test. Sample stimulated with IL-12+IL-15 was compared with every other. **(C)** Degranulation activity of CD56^bright^HLA-DR^+^, CD56^bright^HLA-DR^–^, CD56^dim^HLA-DR^+^ and CD56^dim^HLA-DR^−^ subsets after 24 h incubation with IL-2 and 2 μg/ml sonicate in response to K562 target cells. Data are Mean ± SEM of three independent experiments. Statistical difference was evaluated by two-way ANOVA. *p < 0,05; **p < 0,01, ***p < 0,001.

In PBMC cultures of both healthy donors and tuberculosis patients, the highest IFNγ response to 24 h sonicate stimulation was detected among CD56^dim^HLA-DR^+^ NK cells, compared to CD56^dim^HLA-DR^−^ and CD56^bright^HLA-DR^+^ NK cells (**Figure 2A**, **Supplementary Figure 1A**). Inside the CD56^dim^IFNγ^+^ subset, HLA-DR-positive NK cells comprised around 20% of all cytokine-producing cells in both healthy donors and TB patients (**Supplementary Figure 1B**). In CD56^bright^ NK cell subset, HLA-DR-positive cells also tended to produce IFNγ more actively, but the difference with the respective HLA-DR^−^ NK cells was not statistically significant. At the same time, in isolated NK cell cultures, 24 h incubation with IL-12+IL-15 and sonicate inhibited IFNγ production in HLA-DR^+^ and HLA-DR^−^ cells from both CD56^bright^ and CD56^dim^ fractions, compared to the samples stimulated with cytokines alone (**Figure 2B**). A dose-dependent decrease in the amount of IFNγ-producing cells was registered in a wide range of sonicate concentrations - from 0,25 to 10 μg/ml. Still, CD56^dim^HLA-DR^+^ NK cells responded significantly higher than CD56^dim^HLA-DR^−^ cells, at the level comparable with the CD56^bright^ subset which is generally considered cytokine-productive. Inhibition of cytokine production could be possibly caused by the products of destroyed mycobacteria, and interaction with microenvironment (PBMC) seems to help NK cells overcome the negative effect.

Degranulation activity of isolated NK cells was also significantly suppressed by the presence of mycobacterial sonicate (**Figure 2C** and **Supplementary Figures 2A, B**). When stimulated with IL-2 alone, the response of all studied subsets was comparable, but HLA-DR^+^ NK cells from both CD56^bright^ and CD56^dim^ fractions demonstrated slightly higher degranulation level towards K562 target cells. With the addition of sonicate, all subsets were suppressed, however, CD56^dim^HLA-DR^+^ NK cells were still more effective than their HLA-DR^−^ counterparts (**Figure 2C**).

**Figure 3:**
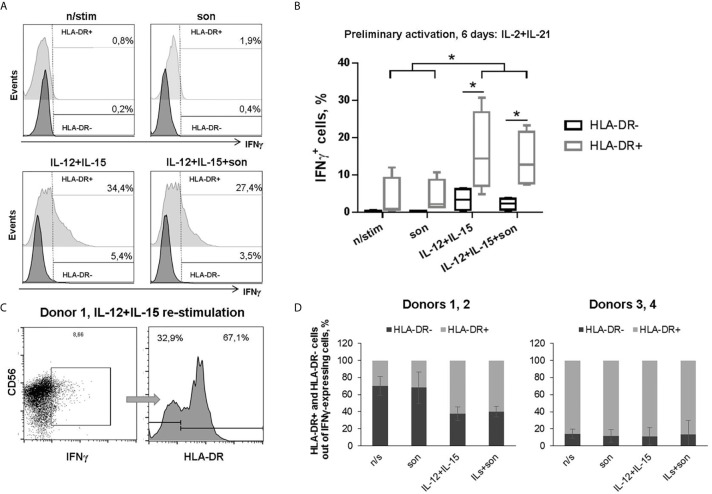
**|** Production of IFNγ by HLA-DR^+^ and HLA-DR^–^ NK cells after 6 days incubation with IL-2+IL-21 and re-stimulation with IL-12+IL-15 and 2 μg/ml sonicate. **(A)** Representative staining and **(B)** summarized data of four independent experiments are shown: median, 10-90% percentile and scatter. Statistical difference was evaluated by paired t-test, *p < 0,05. **(C)** Representative gating of IFNγ-positive NK cells and separating of HLA-DR^+^ and HLA-DR^−^ fractions. **(D)** Fractional composition of the IFNγ-producing NK cells in different donors.

We hypothesized that preliminary activation of primary NK cell cultures can help to overcome the inhibitory effect of destroyed mycobacteria. In another series of experiments, isolated NK cells were first activated with IL-2+IL-21 for 7 days to stimulate their functional activity and increase HLA-DR expressing subset (15, 19, 24), and then re-stimulated with *M. tuberculosis* sonicate and IL-12+IL-15 or IL-2 for functional tests. After IL-2+IL-21 activation, NK cells highly upregulated CD56 (CD56^hi^) and HLA-DR expression (**Supplementary Figures 3A, B**, **4A**), so we could no longer distinguish CD56^bright^ and CD56^dim^ fractions. Increase in CD56 expression in NK cells stimulated with different cytokines and feeder cells has been observed earlier by us and other groups (15, 25, 26). Preliminary activated HLA-DR-expressing NK cells retained IFNγ-producing capacity even in the presence of sonicate (**Figures 3A, B**). Significantly more IFNγ-producing cells were recorded in the HLA-DR^+^ subset comparing to the HLA-DR^–^ subset (**Figure 3B**). Interestingly, when gating on IFNγ-producing NK cells (**Figure 3C**), two different patterns of HLA-DR^+^ and HLA-DR^−^ cells distribution was observed inside this subset: in part of the donors, re-stimulation with IL-12+IL-15 ± son increased the proportion of HLA-DR^+^ cells; while in the other donors, most of the IFNγ-producing cells were HLA-DR-positive regardless of the re-stimulation condition (**Figure 3D**).

**Figure 4:**
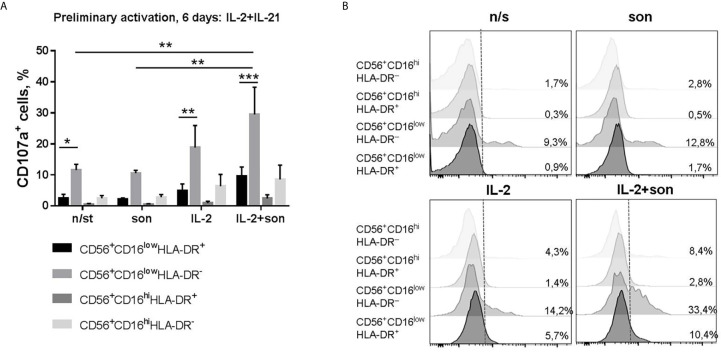
**|** Degranulation activity of CD56^+^CD16^low^HLA-DR^+^, CD56^+^CD16^low^HLA-DR^–^, CD56^+^CD16^hi^HLA-DR^+^ and CD56^+^CD16^hi^HLA-DR^−^ NK cells after 6 days incubation with IL-2+IL-21 and re-stimulation with IL-2 and 2 μg/ml sonicate. **(A)** Summarized data of three independent experiments (Mean ± SEM) and **(B)** representative staining are shown. Statistical difference was evaluated by two-way ANOVA., *p < 0,05; **p < 0,01, ***p < 0,001.

After preliminary activation with IL-2+IL-21, we observed no suppression of degranulation activity in the presence of sonicate (**Figures 4A, B**, **Supplementary Figure 5A**). Moreover, there was a tendency to increased degranulation after re-stimulation with IL-2+son in CD56^hi^CD16^low^ NK cell subset compared to samples re-stimulated with IL-2 alone. Interestingly, the highest degranulation level was registered among CD56^hi^CD16^low^HLA-DR^−^ NK cells, while all HLA-DR-expressing NK cells responded poorly (**Figure 4**). Thus, preliminary stimulation with IL-2+IL-21 upregulates cytotoxic activity mostly in HLA-DR-negative NK cells; further addition of sonicate can increase degranulation response in some cases.

**Figure 5:**
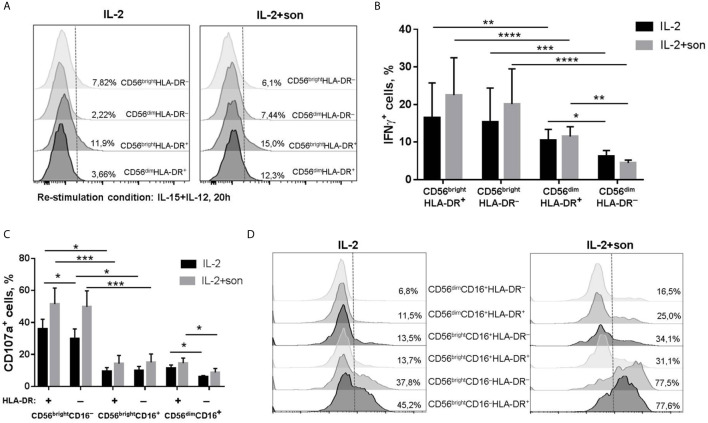
**|** Functional activity of NK cells after 7 day incubation with IL-2 ± sonicate. **(A, B)** Production of IFNγ by CD56^bright^HLA-DR^+^, CD56^bright^HLA-DR^–^, CD56^dim^HLA-DR^+^ and CD56^dim^HLA-DR^−^ subsets after re-stimulation with IL-12+IL-15 for 20h. Representative staining **(A)** and summarized data (Mean ± SEM) of four independent experiments **(B)** are presented. **(C, D)** Degranulation activity of CD56^bright^CD16^−^HLA-DR^+^, CD56^bright^CD16^−^HLA-DR^–^, CD56^bright^CD16^+^HLA-DR^+^, CD56^bright^CD16^+^HLA-DR^–^, CD56^dim^CD16^+^HLA-DR^+^ and CD56^dim^CD16^+^HLA-DR^−^ NK cells after re-stimulation with IL-2 for 20h. Summarized data (Mean ± SEM) of three ndependent experiments **(C)** and representative staining **(D)** are presented. **(B, C)** Statistical difference was evaluated by two-way ANOVA. *p < 0,05; **p < 0,01, ***p < 0,001, ****p < 0,0001.

Experiments were also carried out on prolonged (within 7 days) incubation of NK cells with *M. tuberculosis* sonicate and IL-2, or with IL-2 alone as a control. At the end of this period, cells were put to a rest and then re-stimulated with IL-12+IL-15 or IL-2 for functional tests. After such activation conditions, only part of the NK cells upregulated CD56, so CD56^bright^ and CD56^dim^ fractions were clearly separated (**Supplementary Figures 3C**, **4B**). In degranulation assays, CD16 staining also allowed to differentiate CD56^bright^ NK cells not expressing this receptor (CD56^bright^CD16^−^ fraction), and CD56^bright^ cells which began to express CD16 (CD56^bright^CD16^+^ fraction), moving towards CD56^dim^ phenotype (**Supplementary Figure 5B**). HLA-DR expression was upregulated too, and the proportion of HLA-DR^+^ NK cells was higher in IL-2+son stimulated samples (**Supplementary Figure 3C**), which will be discussed further in phenotype experiments. It was revealed that, first of all, there was no inhibition of IFNγ production or degranulation activity after such prolonged incubation with sonicate. CD56^bright^ NK cells, both HLA-DR^+^ and HLA-DR^−^, produced more IFNγ after re-stimulation than the respective CD56^dim^ NK cells, with a tendency to even higher production after culturing with sonicate (**Figures 5A, B**). In the CD56^dim^ fraction, HLA-DR^+^ NK cells demonstrated higher IFNγ production than their HLA-DR^−^ counterparts, after culturing either with or without sonicate. Altogether, HLA-DR-positive NK cells from both CD56^bright^ and CD56^dim^ subsets demonstrated characteristic cytokine production capacity according to their differentiation stage, shown earlier by us (15), regardless of the sonicate presence. In the degranulation assay, the highest level of CD107a-positive cells was registered in the CD56^bright^CD16^−^ fraction, in both HLA-DR^+^ and HLA-DR^−^ NK cells, again with a tendency to higher response in the cells previously cultured with sonicate (**Figures 5C, D**). In the CD56^dim^ fraction, HLA-DR^+^ NK cells showed slightly higher degranulation activity than their HLA-DR^−^ counterparts, after both culturing conditions. Thus, even prolonged incubation of isolated NK cells with *M. tuberculosis* sonicate induces specific IFNγ response and degranulation activity very poorly and only in the CD56^bright^ fraction, but allows to overcome inhibitory effect of destroyed mycobacteria.

### A Subset of Less Mature, NCR-Expressing HLA-DR^+^ NK Cells Proliferates in Response to *M. tuberculosis* Antigens

**Figure 6:**
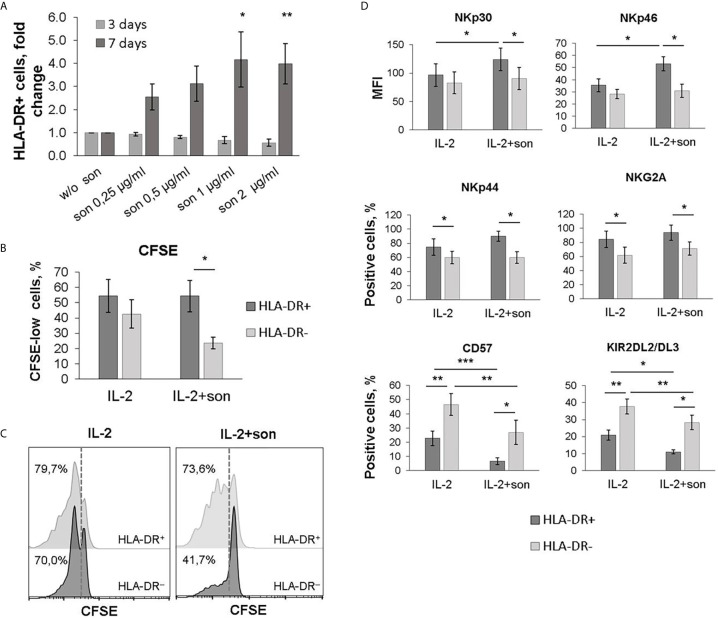
**|** Phenotypic changes during cultivation of NK cells with *M. tuberculosis* sonicate. **(A)** The percentage of HLA-DR-positive NK cells after 3 and 6 days of incubation with IL-2 and sonicate at the indicated concentrations. Data is normalized on the sample without sonicate. Mean ± SD of three independent experiments is presented. Statistical difference with the sample without sonicate was evaluated by paired t-test. **(B, C)** Analysis of the proliferative activity of HLA-DR^+^ and HLA-DR^–^ NK cells upon stimulation with IL-2 ± sonicate by CFSE dilution. Mean ± SD of five independent experiments **(B)** and representative staining **(C)** are presented. **(D)** Expression of the indicated surface markers on HLA-DR^+^ and HLA-DR^–^ NK cells after 6 days of stimulation with IL-2 ± sonicat. Mean ± SD of five independent experiments is presented. **(B, D)** Statistical difference was evaluated by paired t-test. *p < 0,05; **p < 0,01, ***p < 0,001.

We have revealed a number of changes in NK cell phenotype after seven-day stimulation of NK cells with mycobacterial sonicate. At day 7, an increase in the proportion of HLA-DR-positive NK cells was recorded along with the increase in sonicate concentration in a dose-dependent manner, compared with the samples stimulated with IL-2 alone (**Figure 6A**). The analysis of CFSE staining showed there was significantly more proliferating cells in the HLA-DR^+^ NK cell subset compared to the HLA-DR^–^ subset in response to sonicate (**Figures 6B, C**). Thus, recognition of mycobacterial antigens seems to trigger the expansion of HLA-DR-expressing NK cells.

A higher proportion of NKp44^+^ and NKG2A^+^ cells, higher expression level of NKp46 and NKp30 receptors, and a reduced proportion of CD57^+^ and KIR2DL2/DL3^+^ cells was recorded among the HLA-DR-positive NK cell subset after 7 days of stimulation with *M. tuberculosis* sonicate (**Figure 6D**, **Supplementary Figure 6**). Changes in the level of NKp44^+^ and NKG2A^+^ NK cells were similar to those observed in the samples stimulated with IL-2 alone, and are characteristic of activated NK cells in general (27, 28). According to the obtained results, the majority of HLA-DR^+^ NK cells which expanded in response to sonicate exhibited less mature phenotype NKG2A^+^CD57^−^KIR2DL2/DL3^−^, and expressed more NKp46 and NKp30 activating receptors on their surface. It has been shown earlier that NKp46 receptor allows NK cells to recognize macrophages infected with *M. tuberculosis*  *(* 7), so the described NK cell subset may be functionally useful *in vivo*. The less mature phenotype facilitates quick proliferation of this subset in response to further stimulation.

### HLA-DR^+^ NK Cells Mediate Mtb-Induced CD4^+^ T Cell Activation *In Vitro*

**Figure 7:**
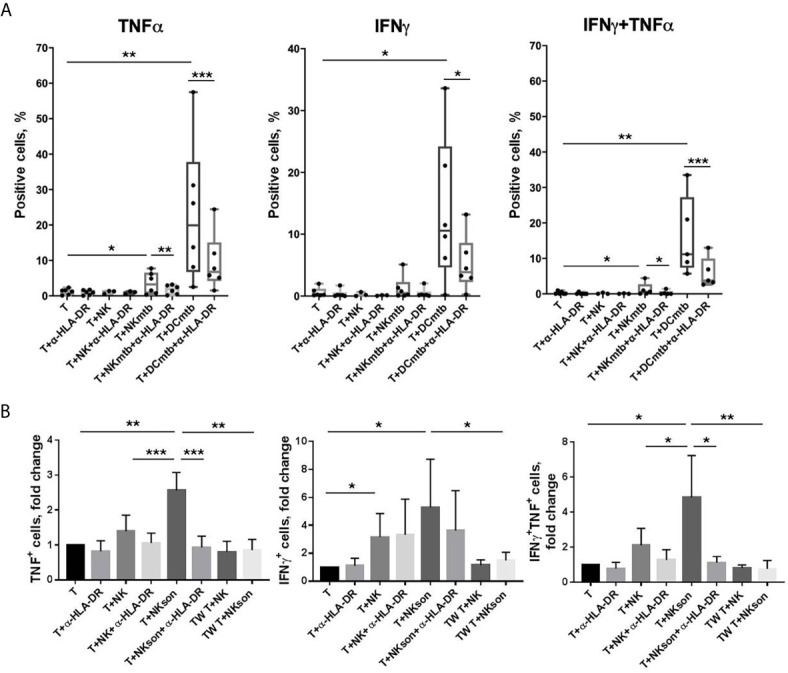
**|** *M. tuberculosis*-induced activation of CD4^+^ T cells by autologous NK cells *in vitro*. **(A)** Production of IFNγ and TNF by T cells measured after 20 h incubation with anti-HLA-DR antibody (α-HLA-DR), NK cells pre-incubated and not pre-incubated with M. tuberculosis sonicate (NK and NKmtb, respectively), and dendritic cells pre-incubated with M. tuberculosis sonicate (DCmtb) in the indicated combinations. Summarized data of six independent experiments (median, 10-90% percentile and scatter) are shown. **(B)** Production of IFNγ and TNF by T cells measured after 20 h incubation with anti-HLA-DR antibody (α-HLA-DR), NK cells pre-incubated and not pre-incubated with M. tuberculosis sonicate (NK and NKmtb, respectively) in simple plates and in the Transwell (TW) in the indicated combinations. Summarized data of three independent experiments (Mean ± SD) are shown. **(A, B)** Statistical difference was evaluated by paired t-test. *p < 0,05; **p < 0,01, ***p < 0,001.

Based on the published data describing antigen-specific activation of T cells by HLA-DR-expressing NK cells (20–22), we hypothesized that HLA-DR^+^ NK cells may also be able to process and present certain mycobacterial antigens, as long as this subset expands in response to sonicate and is shown to accumulate in TB-infected lungs *in vivo* (17, 18). In a series of experiments, NK cells were firstly pre-activated *in vitro* for 10 days in the presence of IL-2, IL-21, and IL-18 to increase the expression of HLA-DR and CD86 (15, 19, 29) (**Supplementary Figure 7**), then part of them was incubated for 24 h with *M. tuberculosis* sonicate (NKmtb) and then co-cultured with autologous CD4^+^ T cells. Autologous dendritic cells pre-incubated with sonicate (DCmtb) and NK cells without preliminary incubation (NK) were added to T cells as control samples. A blocking antibody against HLA-DR was added to certain samples. Part of M. tuberculosis-treated and untreated NK cells were co-cultured with T cells in Transwell plates. After 20 h, T cell activation was assessed by the intracellular levels of IFNγ and TNF – cytokines, characteristic of Th1 type of the response. We have shown that NKmtb induced a statistically significant increase in cytokine production in CD4^+^ T cells, compared to T cells cultured alone and with untreated NK cells, as well as to T cells from Transwell plates (**Figures 7A, B**, **Supplementary Figure 8**). However, the observed effect was significantly lower than in the samples with professional APCs, dendritic cells. It is important to note that after incubation with NKmtb, a double-positive subset of TNF^+^IFNγ^+^ cells appeared among T cells (**Supplementary Figure 8**), which was also clearly recorded in samples with DC and is characteristic of the antigen-dependent functional response (30, 31). Moreover, in both T+DC and T+NKmtb samples, the addition of the HLA-DR-blocking antibody led to a decrease in the production of both cytokines by T cells (**Figure 7A**). Thus, we have registered M.tuberculosis-induced cytokine response of CD4^+^ T cells, which was dependent on cell-to-cell contact with NK cells, pre-incubated with mycobacterial sonicate, and involved HLA-DR molecules. The obtained data indirectly supports the hypothesis that NK cells are able to present certain antigens of *M. tuberculosis* to CD4^+^ T cells and induce their Th1-like polarization.

## Discussion

Several lines of evidence indicate that NK cells may play a significant role at different stages of *M. tuberculosis* infection (32). In humans, a number of clinical studies have explored the potential association between peripheral blood NK cell counts and resistance or susceptibility to tuberculosis (33–35). A reduction in frequency and functionality of CD56^bright^ NK cells has been observed in patients with active tuberculosis and, reciprocally, high blood levels of NK cells were associated with protection in tuberculosis-resistant individuals. At local infection site, NK cells are shown to be present in the TB pleural fluid but not in the pleural fluid of patients with other pathologies (17) and may infiltrate both early and late human lung granulomatous lesions during active TB (9).

A couple of studies have shown that a significant part of NK cells infiltrating TB-infected lungs are expressing HLA-DR – a type of MHC class II (17, 18). In this work, we have revealed that HLA-DR-positive cells are also expanded among CD56^bright^CD3^−^ and CD56^+^CD3^+^ subsets in the peripheral blood of the individuals primarily infected with lung tuberculosis (**Figure 1A**). Considering the reduced content of CD3^+^CD56^−^ T cells and CD3^−^CD56^−^HLA-DR^+^ cells observed by us in these patients, HLA-DR^+^ NK cells, which often display activated phenotype and functions both in normal conditions and during pathology (36), may compensate for certain deficiencies of the adaptive anti-mycobacterial response.

One of the important aspects is that NK cells are able to recognize mycobacteria directly. NK cells from healthy donors respond to stimulation by Bacillus Calmette-Guerin (BCG) *in vitro* by expressing activation markers, increasing cytotoxic activity and cytokine production in the absence of monocytes/macrophages in the system (4–6). Further studies showed that, most likely, NK cells interact with the components of the bacterial cell wall through the activating receptors NKp44 and TLR2 (7, 8). Thus, we tested functional response of HLA-DR^+^ NK cells from healthy donors to the direct stimulation with ultrasonically destroyed *M. tuberculosis* – sonicate. According to the obtained data, *ex vivo* in the presence of other immune cells (PBMC culture) CD56^dim^HLA-DR^+^ NK cells appeared to be the most responsive to sonicate stimulation, although the intensity of the observed IFNγ response varied between donors (**Figure 2A**). It was previously shown that the sensitivity of NK cells to the presence of mycobacteria can differ between individuals (6, 8), which may be dependent from KIR receptor haplotype (37). In the peripheral blood, HLA-DR-expressing CD56^dim^ NK cells may be one of the “sensors” of Mtb antigens, possibly through the interaction with other cells (3), or directly through TLR2 expression (38). In isolated NK cell cultures and without prior stimulation, mycobacterial sonicate had inhibitory effect on NK cell functionality (**Figures 2B, C**), most likely due to virulence factors released after the destruction of bacterial cells. Still, HLA-DR-positive NK cells showed higher resistance to inhibitory effect of sonicate in both CD56^dim^ and CD56^bright^ subsets, with more profound difference in the CD56^dim^ fraction (**Figures 2B, C**). Preliminary activation with IL-2+IL-21 restored IFNγ-producing capacity in HLA-DR^+^ NK cells, and degranulation activity in HLA-DR^−^ NK cells. After prolonged culturing with sonicate and re-stimulation with cytokines, inhibitory effect was not observed too, and the highest functional response was shown by CD56^bright^ NK cells, slightly prevailing in the CD56^bright^HLA-DR^+^ fraction. Thus, NK cells needed either contact with the microenvironment (PBMC), or preliminary activation with cytokines, or re-stimulation after initial contact with mycobacteria to fully conduct their functions.

Direct contact with *M. tuberculosis* sonicate in the presence of IL-2 stimulated an expansion of HLA-DR^+^ NK cell subset during 7 days of *in vitro* culture (**Figures 6A–C**). Evans et al. (6) showed similar response of HLA-DR^+^ NK cells to *in vitro* stimulation with BCG (6). Considering functional data, HLA-DR-expressing NK cell subset may be an important source of IFNγ during the antibacterial immune response, due to its expansion in the peripheral blood of TB patients and in response to *M. tuberculosis* sonicate *in vitro*. According to previous publications, NK-cell-mediated IFNγ production can be substantial in case of impairments in the adaptive immune response against *M. tuberculosis*, in particular, lack of specific CD4^+^ and CD8^+^ T cells (39, 40). IFNγ secreted by NK cells can stimulate many effector mechanisms in macrophages, including the production of reactive oxygen and nitrogen species, or an increase in the expression of the FcγRI receptor for more efficient recognition and phagocytosis of opsonized cells (41, 42).

Phenotypic analysis of the HLA-DR^+^ NK cells expanded in response to IL-2 and *M. tuberculosis* sonicate have shown that most of this subset is comprised of less differentiated NKG2A^+^CD57^−^KIR2DL2/DL3^−^ cells with slightly increased expression of NKp46 and NKp30 activating receptors (**Figure 4C**). According to the literature, NKp46 is able to recognize macrophages infected with *M. tuberculosis* (7). NKp30 expression was shown to be upregulated after NK cell contact with *M. tuberculosis*-infected monocytes, but had no effect on their lysis by NK cells (43). The formation of such subset of less differentiated, but activated NK cells with increased expression of natural cytotoxicity receptors (NCRs) may be characteristic of the first contact with *M. tuberculosis*. Then, hypothetically, NK cells can steadily become more differentiated, increasing their cytolytic potential along with the progression of the infection. Previously, it was shown that BCG vaccination and re-vaccination leads to the development of long-lived pools of CD56^dim^ and CD56^bright^ NK cells with BCG-specific memory-like responses, producing IFNγ (44, 45). Expanded CD56^dim^NKG2C^+^ NK cells have been registered in a cohort of people with latent TB infection (46), suggesting that the presence of this subset is associated with the infection control. Recently, we have shown that CD56^dim^CD57^−^NKG2C^+^ subset, which retains a high level of natural cytotoxicity, has an increased proliferative potential and is a possible precursor of the adaptive NK cells (47). Interestingly, this highly functional subset, like NKG2C-positive NK cells in general, is characterized by an increased level of HLA-DR expression (22, 47).

At last, we have demonstrated that HLA-DR^+^ NK cells are able to mediate mycobacteria-induced and HLA-DR-dependent CD4^+^ T cell activation, assessed by TNF and IFNγ production. (**Figure 7**) These data indirectly supports the hypothesis that NK cells, pre-incubated with *M. tuberculosis*, can induce an antigen-dependent response in CD4^+^ T cells, that is, conduct antigen presentation. Although T cell response induced by Mtb-treated NK cells was rather low comparing to the response induced by dendritic cells in the control samples, these T-NK cell interactions can be important for activating memory T cells at local inflammation cites – for tuberculosis infection, in TB pleural lesions and regions around granulomas. As we already mentioned above, HLA-DR^+^ NK cells are shown to infiltrate TB-infected lungs (17, 18); moreover, the described cells are predominantly CD56^bright^, and this NK cell subset is able to circulate and enter regional lymph nodes, where it can possibly interact with naïve T cells too. The results obtained by us supplement the already published data describing specific activation of T cells by NK cells preincubated with various antigens: tetanus toxin and the allergen of the house dust mite Der pI (20), HSV particles and its glycoproteins (21), HCMV particles and HCMV-antibody complexes (22). Parts of the destroyed *M. tuberculosis* (sonicate) can be recognized and internalized by NK cells *via* TLR2 receptor, similarly to the fact described by Kim et al. for HSV particles (21), as long as NK cells are able to interact with the components of the bacterial cell wall through TLR2 (8). Moreover, cytokine-activated NK cells upregulate NKp44 (27), which is also involved in the direct *M. tuberculosis* recognition (7). Altogether, considering the IFNγ-producing activity and phenotypic changes of the HLA-DR-expressing NK cells in response to interaction with destroyed *M. tuberculosis*, and, above all, the ability to present mycobacterial antigens, pre-activated *in vitro* HLA-DR^+^ NK cells may be considered a subset of potential interest to be targeted by anti-tuberculosis therapy.

## Data Availability Statement

The raw data supporting the conclusions of this article will be made available by the authors, without undue reservation.

## Ethics Statement

The studies involving human participants were reviewed and approved by Ethics committees of the Russian State Medical University and the Central Tuberculosis Research Institute of Russian Academy of Sciences. The patients/participants provided their written informed consent to participate in this study.

## Author Contributions

SK contributed to the study design, conducted main experiments, analyzed data and wrote the manuscript. MS contributed to the functional studies and data analysis. AP analyzed data obtained in TB patients. NK recruited patients and analyzed clinical data. IL contributed to the experimental design and manuscript editing. AS provided expertise. EK contributed to the research conception and design and manuscript editing. All authors contributed to the article and approved the submitted version.

## Funding

This work was supported by Russian Science Foundation, grant #19-15-00439.

## Conflict of Interest

The authors declare that the research was conducted in the absence of any commercial or financial relationships that could be construed as a potential conflict of interest.
