# Supplementary figures and images for: HLA-DR-Positive NK Cells Expand in Response to Mycobacterium Tuberculosis Antigens and Mediate Mycobacteria-Induced T Cell Activation
Source: Front Immunol. 2021 May 3;12:662128. doi: 10.3389/fimmu.2021.662128 (PMC8128146; doi:10.3389/fimmu.2021.662128)

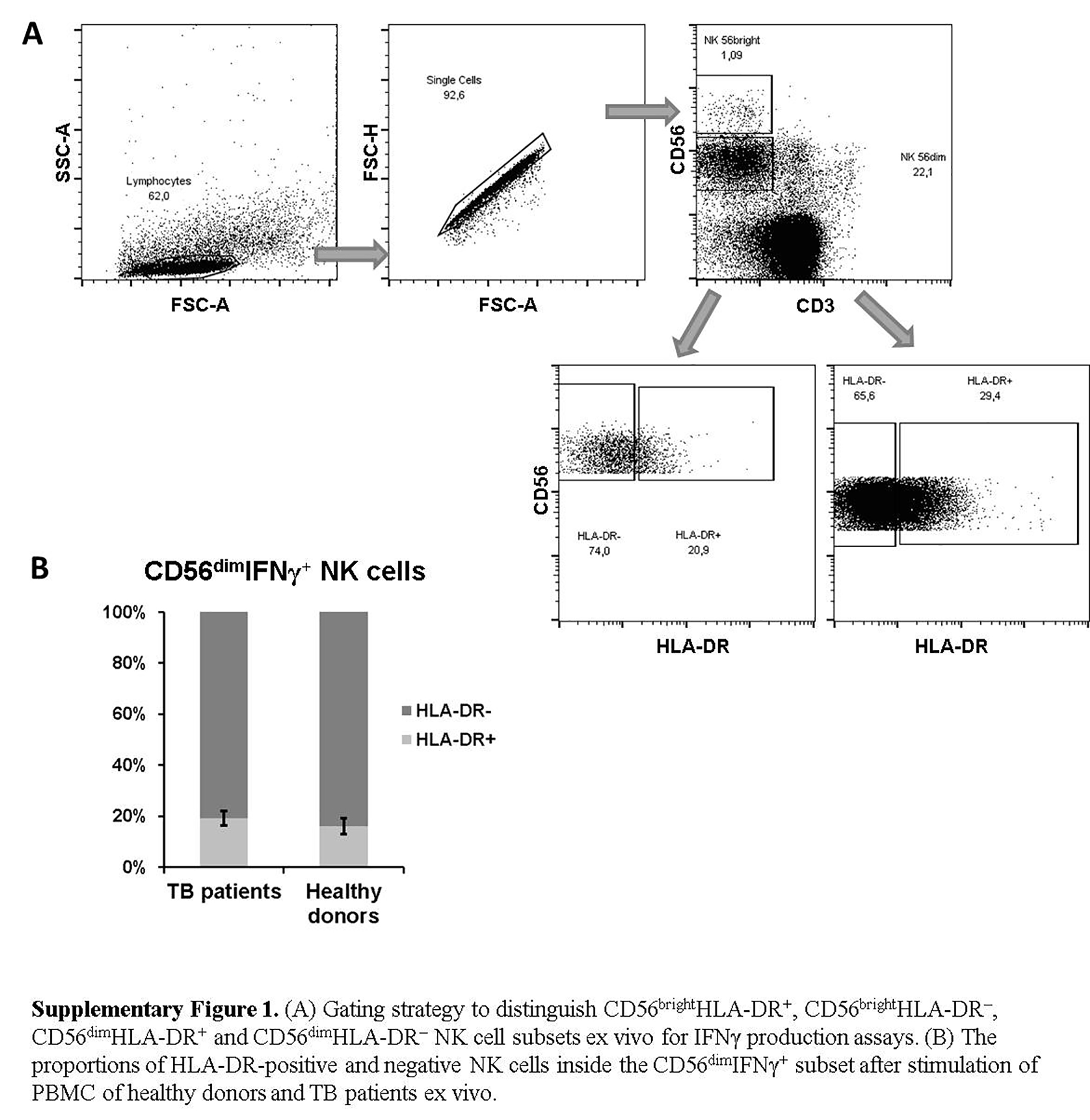

Supplement: Supplementary file 1 [file Image_1.jpeg]

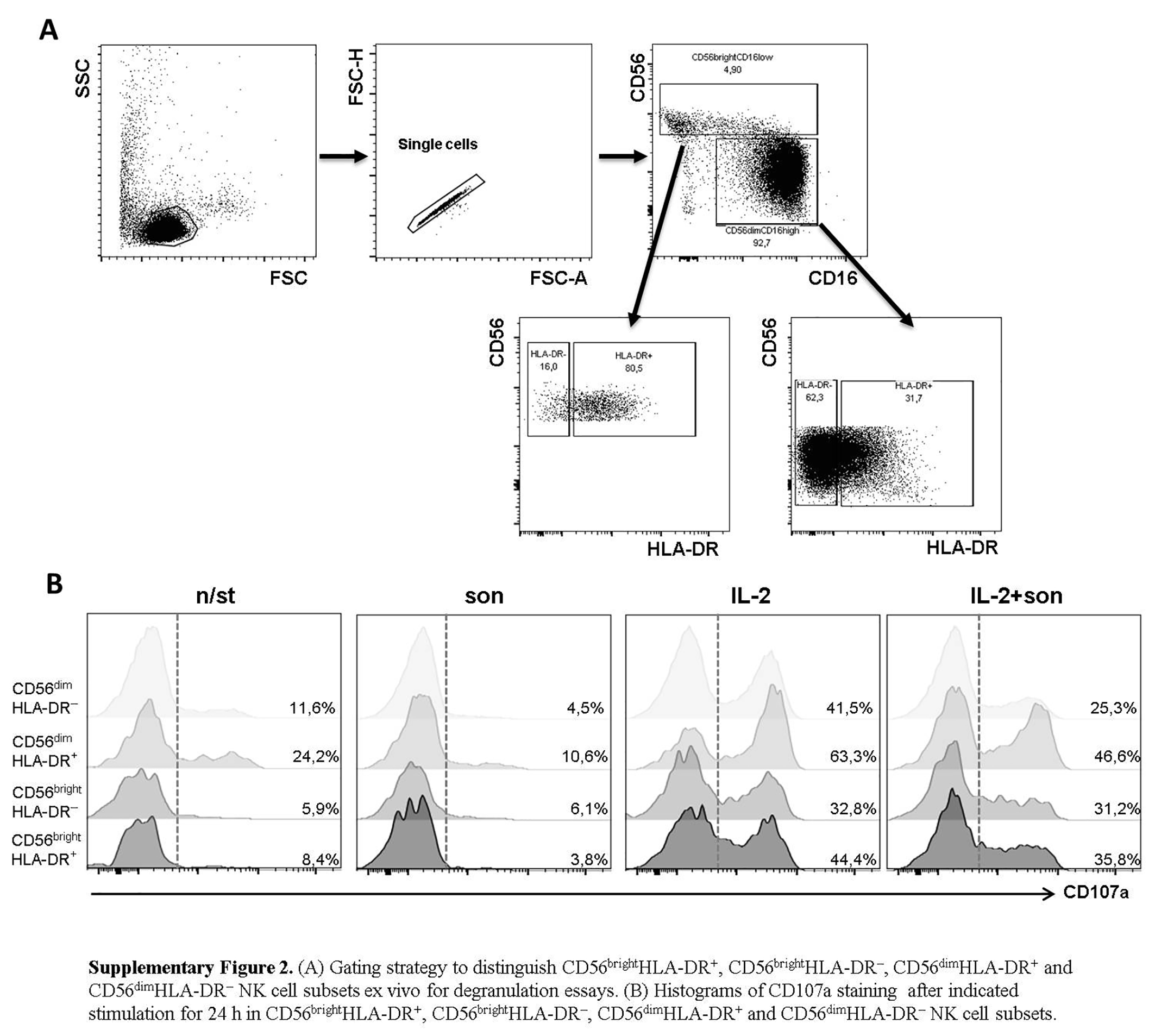

Supplement: Supplementary file 2 [file Image_2.jpeg]

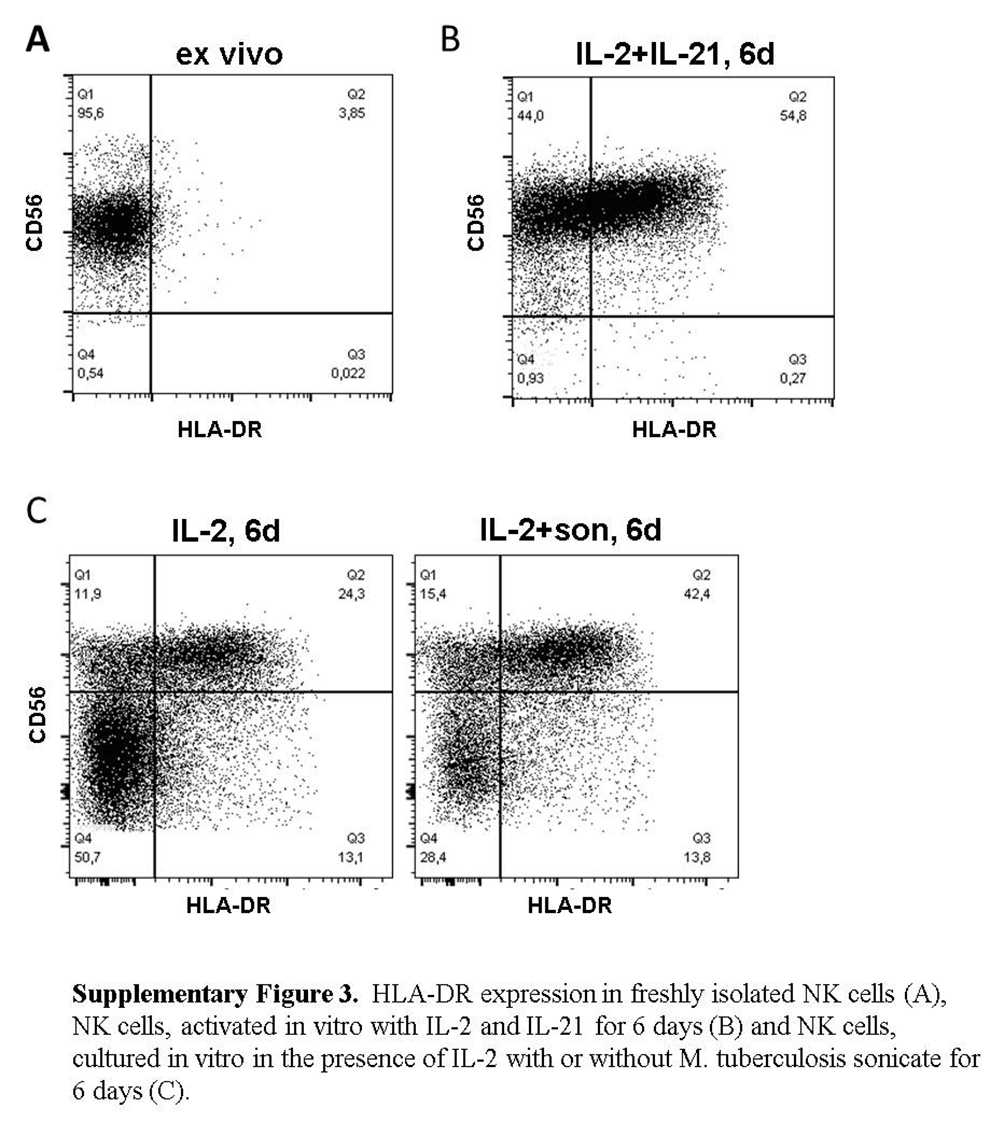

Supplement: Supplementary file 3 [file Image_3.jpeg]

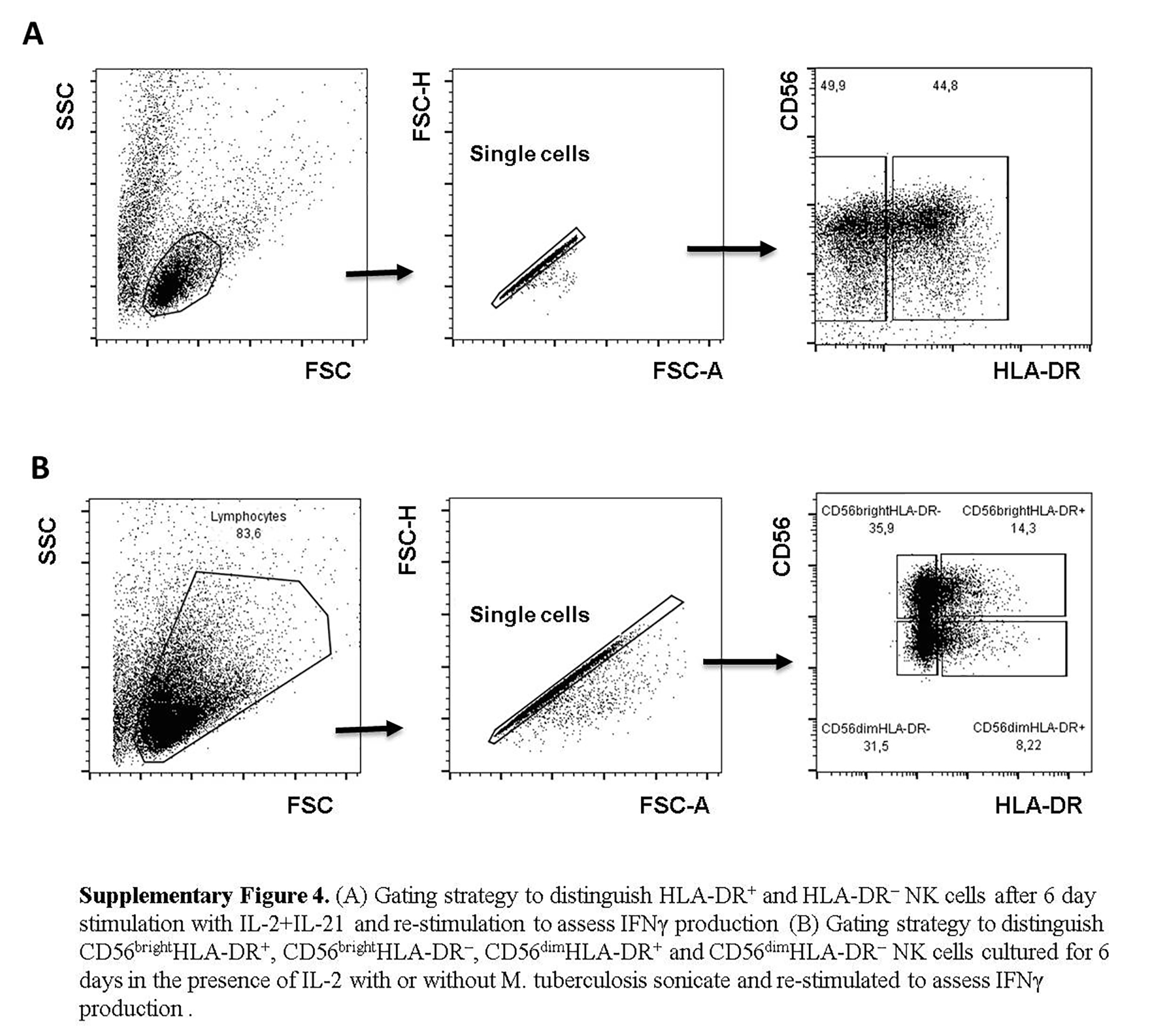

Supplement: Supplementary file 4 [file Image_4.jpeg]

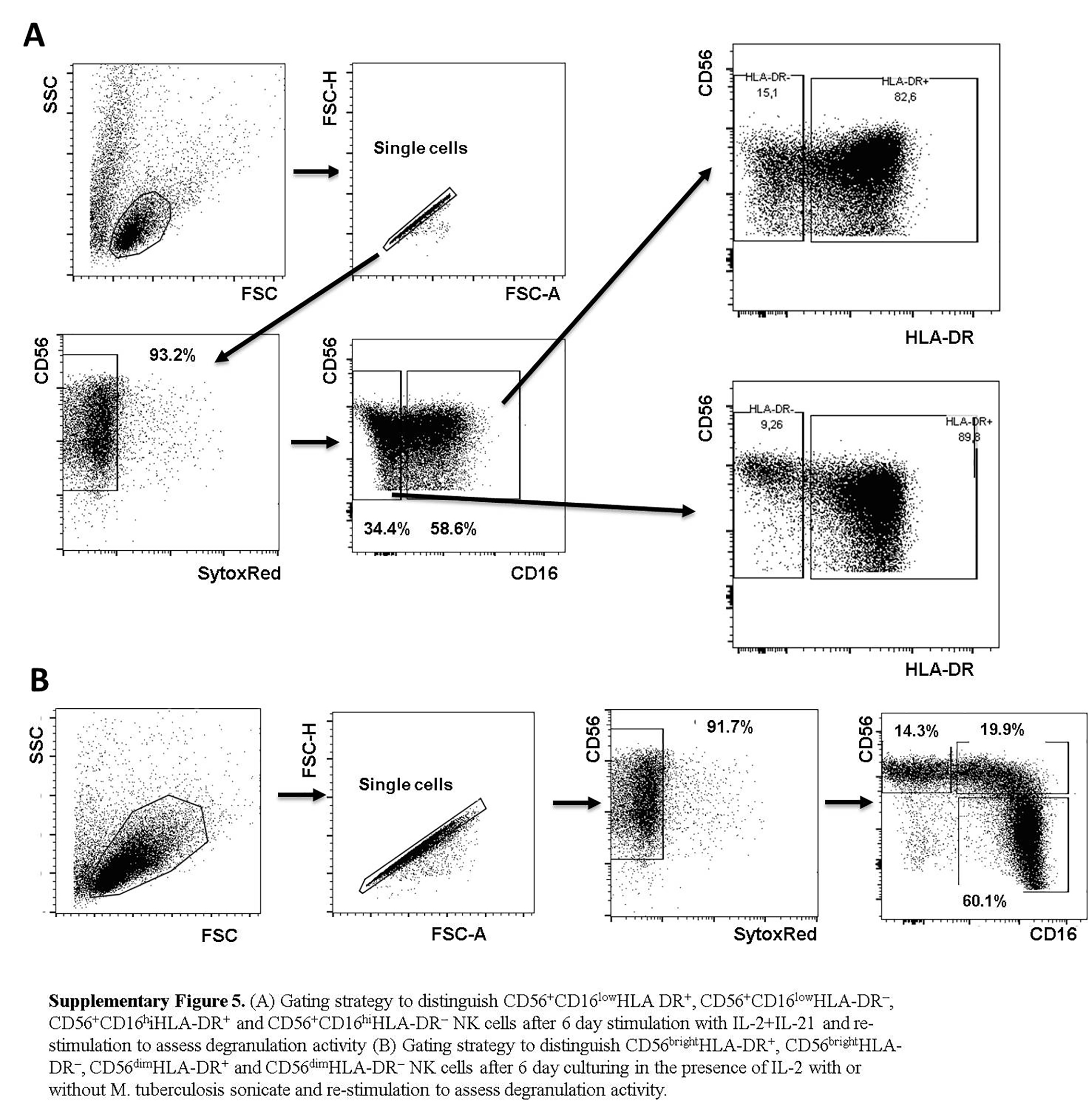

Supplement: Supplementary file 5 [file Image_5.jpeg]

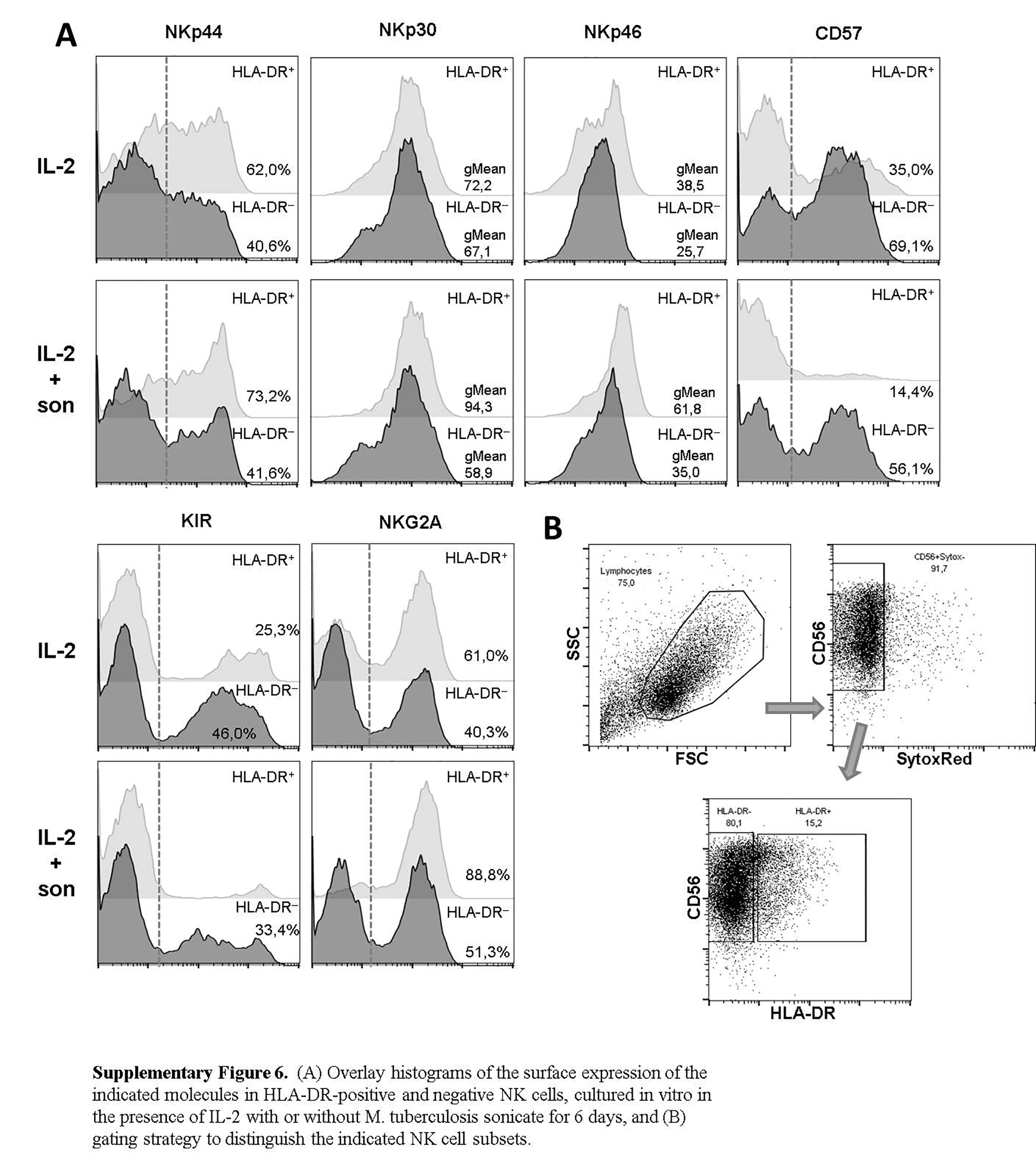

Supplement: Supplementary file 6 [file Image_6.jpeg]

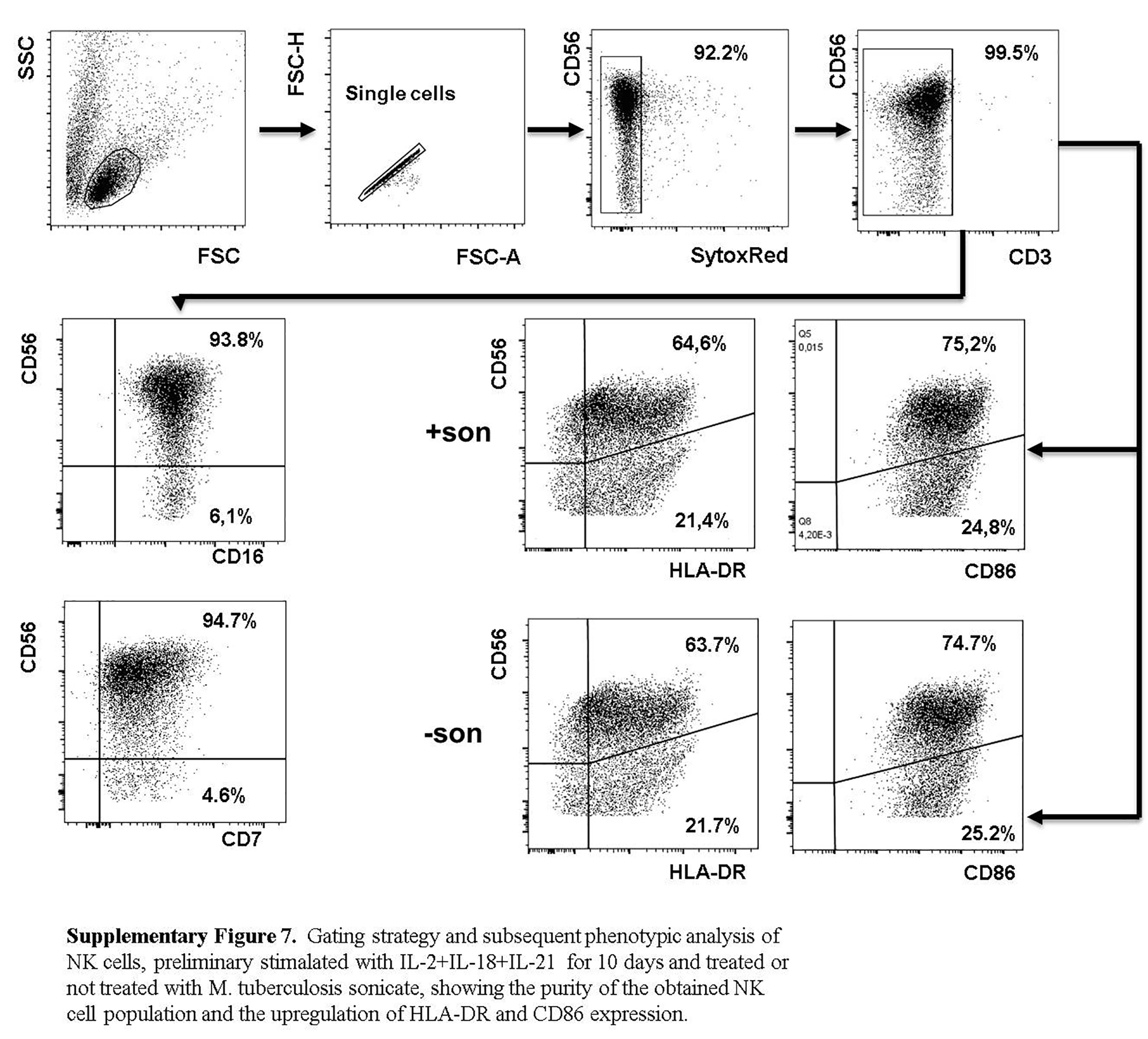

Supplement: Supplementary file 7 [file Image_7.jpeg]

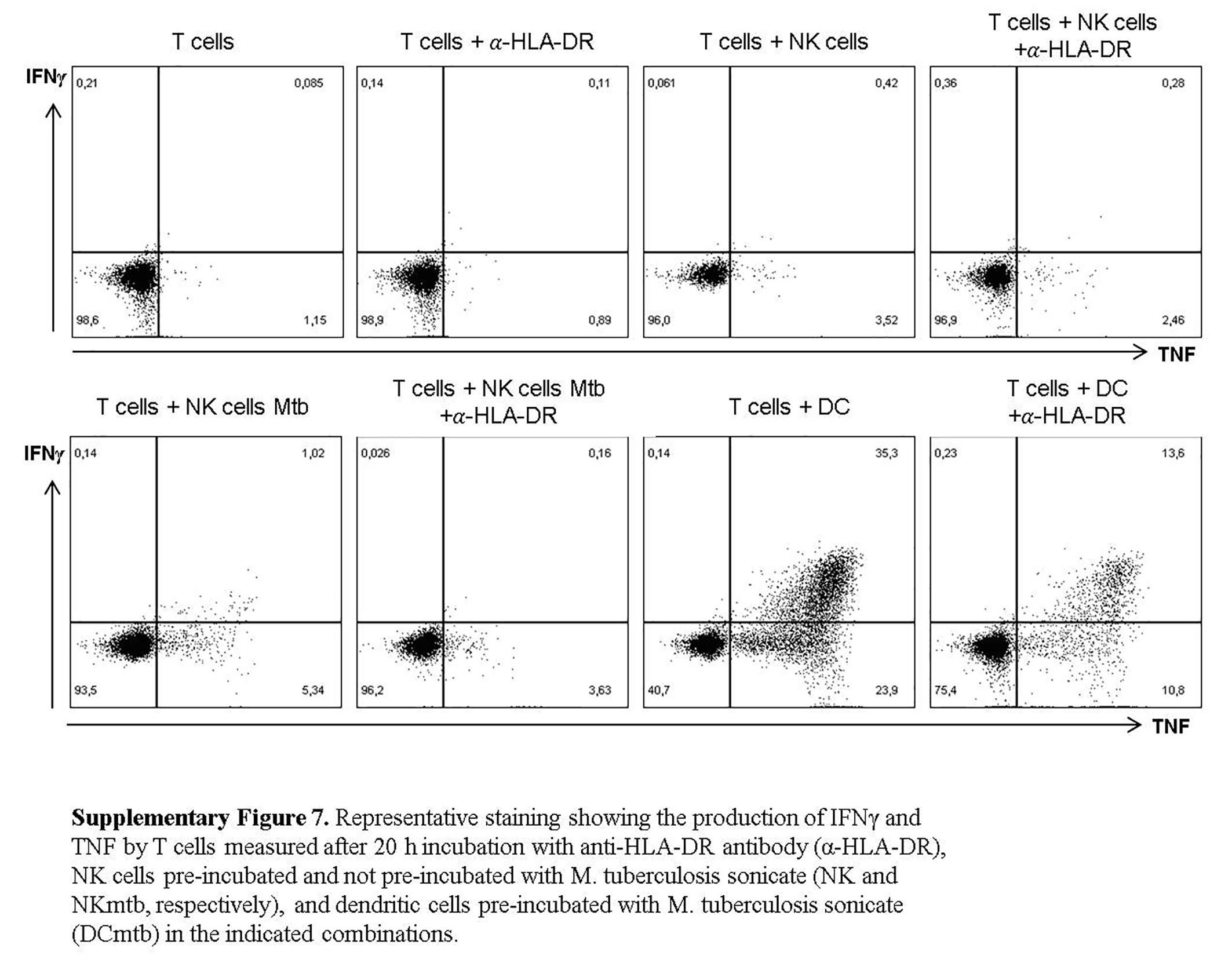

Supplement: Supplementary file 8 [file Image_8.jpeg]
