# Supplementary material for: HLA-DR-Positive NK Cells Expand in Response to Mycobacterium Tuberculosis Antigens and Mediate Mycobacteria-Induced T Cell Activation
Source: Front Immunol. 2021 May 3;12:662128. doi: 10.3389/fimmu.2021.662128 (PMC8128146; doi:10.3389/fimmu.2021.662128)
Supplement: Supplementary file 9 [file Table_1.docx]

| Antigene | Fluorochome | Isotype | Clone | Manufacture |
| --- | --- | --- | --- | --- |
| СD56 | APC | IgG1 | N901 | Beckman Coulter, USA |
| CD56 | PE | IgG1 | N901 | Beckman Coulter, USA |
| CD3 | FITC | IgG1 | UCHT1 | Beckman Coulter, USA |
| HLA-DR | PE-Cy7 | IgG1 | Immu-357 | Beckman Coulter, USA |
| CD8 | PE-Cy5 | IgG1 | B9.11 | Beckman Coulter, USA |
| CD57 | PE | IgM | TB01 | eBioscience, USA |
| NKG2D | PE | IgG1 | CX5 | eBioscience, USA |
| CD3 | PE | IgG1 | UCHT1 | Dako, Denmark |
| NKG2C | AF488 | IgG1 | 134591 | R&D Systems, USA |
| NKG2C | PE | IgG1 | 134591 | R&D Systems, USA |
| KIR2DL2/DL3 | PE | IgG2a | DX27 | Miltenyi Biotec, Germany |
| NKp30 | FITC | IgG1 | P30-15 | Biolegend, USA |
| CD86 | PE | IgG1 | BU63 | Biolegend, USA |
| HLA-DR | FITC | IgG2a | L243 | Sony Biotechnology, USA |
| HLA-DR | PE | IgG2a | L243 | Sony Biotechnology, USA |
| HLA-DR | Brilliant Violet 421 | IgG2a | L243 | Sony Biotechnology, USA |
| HLA-DR | Purified | IgG2a | L243 | Sony Biotechnology, USA |
| IFN-γ | FITC | IgG1 | REA600 | Miltenyi Biotec, Germany |
| IFN-γ | PE | IgG1 | REA600 | Miltenyi Biotec, Germany |
| TNF-α | FITC | IgG1 | REA656 | Miltenyi Biotec, Germany |
| CD4 | APC | IgG1 | REA623 | Miltenyi Biotec, Germany |
| CD14 | PE-Cy7 | IgG1 | REA599 | Miltenyi Biotec, Germany |
| CD16 | VioGreen | IgG1 | REA423 | Miltenyi Biotec, Germany |
| CD107a | VioBlue | IgG1 | REA792 | Miltenyi Biotec, Germany |
